# Dietary (*n-6 : n-3*) Fatty Acids Alter Plasma and Tissue Fatty Acid Composition in Pregnant Sprague Dawley Rats

**DOI:** 10.1100/2012/851437

**Published:** 2012-03-12

**Authors:** Amira Abdulbari Kassem, Md Zuki Abu Bakar, Goh Yong Meng, Noordin Mohamed Mustapha

**Affiliations:** ^1^Department of Veterinary Preclinical Sciences, Faculty of Veterinary Medicine, Universiti Putra Malaysia, 43400 UPM Serdang, Selangor, Malaysia; ^2^Department of Animal Production, Faculty of Agriculture, University of Sana'a, P.O. Box 12426, Sana'a, Yemen; ^3^Department of Pathology and Microbiology Veterinary, Faculty of Veterinary Medicine, Universiti Putra Malaysia, 43400 UPM Serdang, Selangor, Malaysia

## Abstract

The objective of this paper is to study the effects of varying dietary levels of *n-6 : n-3* fatty acid ratio on plasma and tissue fatty acid composition in rat. The treatment groups included control rats fed chow diet only, rats fed 50% soybean oil (SBO): 50% cod liver oil (CLO) (1 : 1), 84% SBO: 16% CLO (6 : 1), 96% SBO: 4% CLO (30 : 1). Blood samples were taken at day 15 of pregnancy, and the plasma and tissue were analyzed for fatty acid profile. The *n-3* PUFA in plasma of Diet 1 : 1 group was significantly higher than the other diet groups, while the total *n-6* PUFA in plasma was significantly higher in Diet 30 : 1 group as compared to the control and Diet 1 : 1 groups. The Diet 1 : 1 group showed significantly greater percentages of total *n-3* PUFA and docosahexaenoic acid in adipose and liver tissue, and this clearly reflected the contribution of *n-3* fatty acids from CLO. The total *n-6* PUFA, linoleic acid, and arachidonic acid were significantly difference in Diet 30 : 1 as compared to Diet 1 : 1 and control group. These results demonstrated that the dietary ratio of *n-6 : n-3* fatty acid ratio significantly affected plasma and tissue fatty acids profile in pregnant rat.

## 1. Introduction

Fatty acids perform two major physiological roles in mammalian tissues: a structural role and a role in energy storage and production. First, fatty acids are the units of phospholipids and glycolipids and, therefore, important constituents of biological membranes. In fact, the fatty acid chains are more than half the mass of most major phospholipids and they are primarily responsible for the apolar nature of the membrane bilayer [1]. The composition of dietary fat is extremely important in the metabolism of PUFA in body tissues, because each dietary fatty acid will influence the utilization of other fatty acids [2]. Linoleic acid, ALA, and oleic acid (nonessential) are competitive substrates for the same sequence of desaturation enzymes. The affinity of these fatty acids for the desaturation enzymes is as follows: ALA > LA > oleic acid. Low concentrations of ALA are very effective in suppressing the metabolism of LA. However, moderate levels of LA are necessary to inhibit the metabolism of ALA, whereas only high concentrations of oleic acid can suppress the metabolism of LA. Consequently, the metabolites of ALA and LA are normally found in higher amounts than the metabolites of oleic acid in normal body tissues and fluids. Because the dietary fat composition can be altered in favor of one of the above fatty acids, the metabolism can be shifted according to the affinity, as well as the amount of fatty acid consumed. Therefore, dietary intake determines to a great extent the fatty acid composition of phospholipids in the plasma, tissues, and cell membranes [3]. The plasma and adipose tissue FA composition reflect the composition of the diet to a large extent, but they also reflect de novo FA synthesis coupled with chain elongation and desaturation [4]. This process occurs in the liver, mammary gland, and adipose tissue. Fatty acids can also be altered by desaturation or elongation reactions, which mainly occur in the liver [5]. Numerous studies have associated alterations in dietary fats of maternal diet during pregnancy or during weaning period with alterations in fatty acid composition of cell membranes and organelles in brain and behavioral deficits in rats and mice [6], and the DHA is important in normal brain development and function because it is positively correlates with the changes in cognitive and behavioural performance [7]. The objective of this study was to assess the effect of dietary fatty acids supplementation on plasma and membrane fatty acid composition of the liver and adipose tissues.

## 2. Materials and Methods

### 2.1. Animals and Experimental Design

Twenty-eight (28) female, two-month-old Sprague-Dawley (240 ± 20 g body weight) rats were used in this experiment. After 2 weeks of adaptation, the rats were randomly divided into four treatment groups consisting of seven rats in each group. The experiment was approved by Institutional Animal Care and Use Committee (IACUC) of Faculty of Veterinary Medicine Universiti Putra Malaysia. The cod liver oil (Seven seas, Malaysia) was used as the main source of *n-3 *PUFA (Eicosapentaenoic acid (EPA) and docosahexaenoic acid (DHA)) and soybean oil is the main source of *n-6* fatty acids and linolenic acid (LA). The SBO and CLO were first analysed to determine the fatty acid profile and are shown in Table 1. The initial body weight of each rat was recorded, and during the whole experiment the body weight and feed intake of each rat were taken every week. After adaptation period, the rats were randomly assigned to dietary treatments. The treatment groups include rats fed with normal rat chow diet the control (C) group, rats fed chow diet supplemented with 5% (w/w) SBO and 5% (w/w) CLO (Diet 1 : 1 group), rats fed chow diet added with 8.4% (w/w) SBO and 1.6% (w/w) CLO (Diet 6 : 1 group), and rats fed chow diet added with 9.6% (w/w) SBO and 0.4% (w/w) CLO (Diet 30 : 1 group). The rats were fed 7% of body weight daily, and water was provided *ad libitum*. The diets were prepared daily to minimize rancidity and oxidative damage the rats were fed once daily and the leftover feeds were collected before new feeding. After two months of feeding trial, daily vaginal smears were taken and examined immediately to determine the estrous cycle of each rat. The pregnancy was induced by overnight caging of a proestrus female with a male of proven fertility. The next day, the presence of a vaginal plug or spermatozoa in the vaginal smear was termed as day 0 of pregnancy. Pregnant females were separated from the male rats after confirmation of pregnancy. The rat were individually housed in polycarbonate cages (43 × 28 × 16 cm) with sawdust bedding, in controlled room temperature (23 ± 2°C) with 12 h of light and 12 h of darkness. At day 15th of each pregnancy, rats were anesthetized with an intraperitoneal injection of ketamine 60 mg/kg body weight (Narketan Vetoquinol SA, 70204 Lure, Sedex, France) + xylazine 8 mg/kg body weight (Troy Laboratory PTY Ltd, Australia) and blood was collected via cardiac puncture using 26 gauge needle. Before collecting the blood, the syringe was coated with EDTA to prevent blood clotting within the syringe. The blood collected was placed in EDTA and immediately placed on ice. Samples were labeled for identification and centrifuged at 3000 G for 10 min plasma was removed and stored at −80°C until analysed within one week. Thereafter, the rats were immediately sacrificed. In addition, following exsanguinations, about 2 g each of liver and abdominal adipose tissue was collected and kept at −20°C until subsequent analysis.

### 2.2. Fatty Acids Determination

The total fatty acids were extracted from feeds and tissue based on the method of [8], modified by [9], using chloroform: methanol 2 : 1 (v/v) containing butylated hydroxytoluene to prevent oxidation during sample preparation. The experimental diets or tissues were homogenized in 40 mL chloroform : methanol (2 : 1 v/v). The mixture containing the extracted fatty acids was filtered through No. 1 Whatman paper (Whatman International Ltd., Maidstone, UK) into a 250 mL separating funnel using a funnel. Ten (10) mL of normal saline solutions were added to facilitate phase separation. The mixture was then shaken vigorously for one minute and was left to stand for four hours. After complete separation at the end of the fourth hour, the upper phase was discarded and the lower phase was collected in a round bottom flask and evaporated via rotary evaporation (Laborota 4000-efficient, Heidolph, Germany) at 70°C.

The total lipid extract was then immediately transferred into a capped methylation tube by rediluting it with 5 mL fresh chloroform : methanol (2 : 1, v/v). Transmethylation of the extracted fatty acids to their fatty acid methyl esters (FAME) was carried out using 14% methanolic boron trifluoride (BF3) (Sigma Chemical Co., St. Louis, MO, USA) according to methods in AOAC (1990). The internal standard, heneicosanoic acid (21 : 0) (Sigma Chemical Co., St. Louis, Mo, USA), was added to each sample prior to transmethylation to determine the individual fatty acid concentrations within the samples. The methyl esters were quantified by Gas Chromatography (GC) (Agilent 7890 N) using a 30 m × 0.25 mm ID (0.20 *μ*m film thickness) Supelco SP-2330 capillary column (Supelco, Inc., Bellefonte, PA, USA). One microliter of fatty acid methyl ester (FAME) was injected by an autosampler into the chromatograph, equipped with a split/splitless injector and a flame ionization detector (FID). The injector temperature was programmed at 250°C, and the detector temperature was 300°C. The initial column temperature was set at 100°C for 2 min, and then increased at 10°C/min to 170°C where it was held for another 2 min. Finally, it was warmed to 220°C at 7.5°C/min to reach a final temperature of 250°C and held for 20 min to facilitate optimal separation. All results of fatty acid presented as the percentage of total fatty acids.

### 2.3. Data Analysis

Data were analysed as a completely randomized design experiment using the General Linear Model of SAS 9.02 software (Statistical Analysis Systems Institute Inc., 1992). The fatty acid profiles of the rat treatment diets and different tissue were analysed across treatment groups using the one-way analysis of variance (ANOVA) method. Significantly different means were then elucidated using the Duncan's multiple range tests. All statistics were performed at 95% confidence.

## 3. Result

### 3.1. Fatty Acids Profile of Treatment Diets

The fatty acid profiles of treatment diets are summarized in Table 1. These diets contain both the *n-6* (LA) and *n-3* (ALA) essential fatty acids (EFA). Diet 30 : 1 was used to reflect the higher dietary fat intake of a western human diet, while maintaining the relative contributions of LA and ALA observed in the 1 : 1.

Using high level of SO (30 : 1 ratio), the total *n-6* PUFA increased in the Diet 30 : 1 (48.03 g/100 g), while using high level of CLO (1 : 1 ratio), the total *n-3 * PUFA increased in Diet 1 : 1 (7.87 g/100 g). The *n-6* : *n-3 *ratio increased progressively from 4.92 in Diet 1 : 1 to 44.80 in Diet 30 : 1 and 64.29 in Diet Control. Diet 30 : 1 contained highest linoleic acid (18 : 2 *n-6*), which was the major fatty acid, while Diet 1 : 1 contained highest ALA (18 : 3 *n-3*). The major fatty acids content of Diet 6 : 1 was always in between the Diet 1 : 1 and Diet 30 : 1.

### 3.2. Fatty Acids Profile of Plasma

Table 2 showed the plasma fatty acids profiles of the rats treated with different diets after 10 weeks of feeding trial. In the current experiment, 22 fatty acids were detected in plasma. Plasma fatty acids profiles of the Diet 6 : 1 and Diet 30 : 1 groups showed significantly greater (*P* < 0.05) percentages of total *n-6* PUFA as compared to Diet 1 : 1 and Diet Control groups. Arachidonic acid (AA) was significantly higher (*P* < 0.05) in Diet 6 : 1 and Diet 30 : 1 group as compared to Diet 1 : 1 group. Diet 1 : 1 group had the highest value of eicosapentaenoic acid (EPA) and docosahexaenoic acid (DPA) in comparison to Diet 6 : 1 and Diet 30 : 1 groups. The plasma total PUFA *n-3* fatty acid in Diet 1 : 1 group was the highest at 11.21% (*P* < 0.05), and this clearly reflected the contribution of *n-3* fatty acids from CLO. Linoleic acid (LA) was the main unsaturated fatty acid in the plasma of all diet groups. The *n-6* : *n-3 *ratio was significantly lower (3.49%) in the Diet 1 : 1 group (*P* < 0.05) as compared to other groups. Both the Diet 6 : 1 and Diet 30 : 1 groups had almost similar amount (*P* > 0.05) of *n-6* : *n-3 *ratios. The *n-6* : *n-3 *ratio was in the increasing order starting from Diet 1 : 1, Diet 6 : 1, Diet 30 : 1, and Diet Control groups.

### 3.3. Liver Tissue Fatty Acids Composition

Table 3 showed the liver tissue fatty acids profiles of the rats treated with different diets after 10 weeks of feeding trial. The Diet 1 : 1 group demonstrated the lowest *α*-Linolenic acid (ALA, 18 : 3, *n-3*) and highest docosahexaenoic acid (DHA, 22 : 6, *n-3*) contents in the liver lipid fractions examined. There was also a higher content of Ecosapentaenoic acid (EPA; 22 : 5, *n-3*) in the liver lipids of Diet 1 : 1 group as compared to the other groups (Table 3).

The total *n-3* PUFA was significantly higher in the Diet 1 : 1 group as compared to other groups. The total saturated, unsaturated, and monounsaturated fatty acids were not significantly different (*P* > 0.05) among the groups. Total percentage of DHA in the Diet 1 : 1 group was increased as compared to other groups, but not significantly different (*P* > 0.05). The percentage of total *n-6* PUFA was not significantly different (*P* > 0.05) among the groups. No significant differences were observed for the *n-6* : *n-3 *ratio, Unsat : Sat and Poly : Sat ratios among the treatment groups.

### 3.4. Adipose Tissue Fatty Acids Composition

Table 4 showed the adipose tissues fatty acids profiles of the rats treated with different diets after 10 weeks of feeding trial. Dietary fatty acids supplementation had significant effects on the fatty acids composition of the adipose tissue. The Diet 1 : 1 group showed significantly greater (*P* < 0.05) percentages of total *n-3* PUFA and docosahexaenoic acid (DHA) as compared to other groups, and this clearly reflected the contribution of *n-3* fatty acids from CLO. The percentages of total *n-6* PUFA, linoleic acid (LA), and arachidonic acid (AA) were significantly different (*P* < 0.05) in Diet 30 : 1 as compared to Diet 1 : 1 and Diet Control groups, but not significantly different when compared with Diet 6 : 1 group.

In the Diet 1 : 1 group, decreased level of AA was observed as compared to the Diet 30 : 1 group. The total unsaturated fatty acids (UFA) in the Diet Control was significantly decreased (*P* < 0.05) as compared to other treatment groups. Rats fed with Diet 1 : 1 and Diet 6 : 1 had significantly higher adipose total UFA as compared to the control group, but significantly lower than the Diet 30 : 1 group. However, the percentages of Unsat : Sat and Poly : Sat ratio were significantly higher (*P* < 0.05) in the Diet 30 : 1 group as compared to other groups.

## 4. Discussion

Fatty acids composition of a diet is known to influence the fatty acid composition of stored and structural lipids in the body [3]. The current study showed the close link between dietary and tissue fatty acids constitution. This diet contains both *n-6* and *n-3* EFA (LA and ALA). The high dietary *n-6 *: *n-3* ratio soyabean oil diet was used to reflect the higher dietary fat intake of a western human diet, while maintaining the relative contributions of LA and ALA observed in the low dietary *n-6 *: *n-3* ratio soyabean oil diet. Diet had a significant effect on the fatty acid composition of all plasma fatty acids. The low ratio in Diet 1 : 1 (ALA-rich) group had significantly higher AlA content of plasma fatty acids profiles. The content of LC *n-3* PUFA was also affected by diet. The cod liver oil on Diet 1 : 1 groups had the highest EPA content in plasma fatty acids profiles. This effect was not a simple function of the quantity of ALA available, as the high soyabean oil on Diet 6 : 1 and 30 : 1 group had the lowest EPA content, suggesting that the synthesis of LC *n-3* PUFA may be suppressed in rats by a high LA diet but that this can be overcome by increasing the ALA content of the diet. These observations are in accordance with those in human studies, where increased dietary ALA has been demonstrated to result in significantly increased plasma EPA status, but without an associated increase in plasma DHA status [8, 9].

The plasma and adipose tissue fatty acid composition not only reflect the composition of the diet to a large extent, but also reflect de novo fatty acids synthesis coupled with chain elongation and desaturation [4]. The earlier results of this study showed that the CLO contains a higher percentage of total *n-3* PUFA, linolenic acid (ALA, *n-3*), and docosahexaenoic acid (DHA, *n-3*), whereas soybean oil (SO) contains a higher percentage of linoleic acid (LA, *n-6*) and total *n-6* PUFA which agrees with previous studies [10, 11]. Existing data indicates that the fatty acid composition of human plasma lipids is significantly altered during pregnancy [12–14]. Pregnant animals had been identified to have significant effects upon plasma and liver fatty acid composition, including increases in DHA status [15–17]. Thus, the current study confirmed that supplementing dietary fatty acids significantly altered plasma and tissue fatty acid composition, particularly for the DHA and AA. In addition, in this study, the arachidonec acid (AA) content of tissues in pregnancy in the Diet 30 : 1 group was markedly increased, which indicates that maternal long chain *n-6* PUFA (linoleic acid) synthesis is also significantly affected by the pregnancy. The effect of pregnancy upon arachidonic acid status was tissue specific and indicates that this fatty acid may be preferentially mobilised into the maternal plasma in order to be available to the fetus. There was an indication that the linoleic acid (*n-6*) content of plasma fatty acids is higher at day 15 of gestation in the Diet 30 : 1 groups, which may indicate either increased mobilisation of ALA from adipose tissue or lower rates of LC *n-3* PUFA synthesis at this point of gestation.

Dietary *n-3* PUFA in Diet 1 : 1 group had the highest eicosapentaenoic acid (EPA) and docosahexaenoic acid (DPA) content in all plasma fatty acids fractions as compared to other groups, and high level of arachidonic acid (AA) in plasma of Diet 30 : 1 and Diet 6 : 1 groups. This can be explained by the inhibitory effect of cod liver oil on the enzymes involved in the synthesis of AA from linoleic acid (LA). Reference [18] has shown an inhibiting effect of long-chain (LC) *n-3* fatty acids on delta 6 desaturase activities and subsequently reduction in AA level in plasma of rats supplemented with cod liver oil [19] observed a greater integration of EPA and lower levels of arachidonic acid in brain fatty acids in rats fed fish oil and [20], reported incorporation of docosahexaenoic acid (DHA) into cardiac organelles. It is well known that plasma and tissue fatty acids profile followed that of the diet [3, 10], as well as providing precursors or substrates. The *n-3* and *n-6* PUFAs interact and compete with each other for incorporation into phospholipids and as substrates for metabolic enzymes (especially desaturase and COX) [21–23]. Changes in the amounts of PUFAs or their ratios may affect production of PGs in the reproductive system in both cows [24] and humans [25].

Diet supplemented with *n-3* PUFA has been shown to inhibit Δ6 desaturase activity [26]. Potential explanation of the elevation of plasma LA levels with elevated *n-3* PUFA ingestion has been reported previously [27, 28]. In the present study, increasing LA levels in Diet 30 : 1 group led to an increase in plasma LA level. When feeding soybean oil as a source of the arachidonic acid precursor (LA), there was a significant increase in plasma AA content. This increase is in agreement with [10] who feed the rats with high level of safflower oil.

## 5. Conclusion

In this study, diets with higher PUFA *n-6 *: *n-3* ratios resulted in higher AA and lower DHA levels in plasma. It is important to consider that the circulating concentrations of fatty acids such as DHA during pregnancy will be affected by the transfer of fatty acids to the developing fetus in pregnant rat. This study also successfully demonstrated the changes induced by varying levels of dietary *n-6* : *n-3* PUFA ratio on plasma and tissue fatty acid contents in pregnant rat.

## Figures and Tables

**Table 1:** Fatty acid composition (g/100 g total fatty acids) of the treatment diets (*n* = 3).

Fatty acid composition (g/100 g) of feed sample	Group			
	Diet (1 : 1)	Diet (6 : 1)	Diet (30 : 1)	Diet (control)
C14 : 0 myristic acid	2.22	1.03	0.41	1.47
C16 : 0 palmitic acid	14.55	13.43	12.94	16.14
C16 : 1 palmitoleic acid	2.48	1.12	0.40	0.54
C17 : 0 heptadecanoic acid	0.72	1.03	0.53	1.02
C18 : 0 stearic acid	3.99	4.26	4.27	3.73
C18 : 1 oleic acid	25.51	27.08	26.65	26.42
C18 : 2 *n*-6 linoleic acid (LA)	34.88	43.08	47.70	40.75
C18 : 3 *n-*3 linolenic acid (ALA)	0.70	0.54	0.77	0.68
C20 : 0 arachidic acid	3.94	4.87	4.96	3.80
C20 : 4 *n*-6 arachidonic acid	3.83	1.52	0.32	1.95
C20 : 5 *n*-3 ecosapentaenoic acid	3.41	0.94	0.39	Nd
C22 : 6 *n*-3 docosahexaenoic acid	3.77	1.08	0.65	Nd
Total saturated fatty acid	25.42	24.60	23.78	26.15
Total unsaturated fatty acid	74.58	75.40	76.22	73.85
Total MUFA fatty acid	27.99	28.40	27.05	30.46
Total PUFA *n*-3	7.87	2.40	1.15	0.68
Total PUFA *n*-6	38.72	44.60	48.03	42.70
*n*-6 : *n*-3 ratio	4.92	18.20	44.80	64.29
Unsat : Sat	2.93	3.06	3.21	2.86
Poly : Sat ratio	1.83	1.92	2.07	1.67

*∑*SFA = sum of C10 : 0, C12 : 0, C14 : 0, C15 : 0, C16 : 0, C17 : 0, C18 : 0, C20 : 0.

*∑*MUFA = sum of C14 : 1, C16 : 1, C18 : 1.

*∑*PUFA *n*-6 = C18 : 2*n*-6, C20 : 4*n*-6.

*∑*PUFA *n*-3 = C18 : 3*n*-3, C22 : 6*n*-3.

*n*-6/*n*-3 = (C18 : 2*n*-6+ C20 : 4*n*-6)/(C18 : 3*n*-3+ C22 : 6*n*-3).

**Table 2:** Plasma fatty acid composition (mg/100 mL; Mean ± SE; *n* = 7) of rats from different treatment groups after 10 weeks of feeding.

Fatty acid composition(g/100 mL) of plasma	Group			
	Diet (1 : 1)	Diet (6 : 1)	Diet (30 : 1)	Diet (control)
C12 : 0 lauric acid	2.25 ± 0.28^a^	1.02 ± 0.16^b^	1.84 ± 0.09^a^	0.56 ± 0.19^b^
C14 : 0 myristic acid	1.31 ± 0.33^a^	1.19 ± 0.41^a^	Nd	0.53 ± 0.11^b^
C15 : 0 pentadecanoic acid	1.66 ± 0.30^a^	1.67 ± 0.28^a^	1.12 ± 0.19^a^	0.41 ± 0.05^b^
C16 : 0 palmitic acid	15.53 ± 0.80^b^	12.04 ± 0.83^b^	13.75 ± 1.07^b^	21.67 ± 2.11^a^
C16 : 1 palmitoleic acid	0.50 ± 0.05^b^	0.94 ± 0.15^ab^	0.72 ± 0.14^ab^	1.27 ± 0.22^a^
C17 : 0 heptadecanoic acid	14.55 ± 0.36^ab^	14.76 ± 0.70^a^	13.83 ± 0.89^ab^	12.20 ± 0.90^b^
C18 : 0 stearic acid	10.34 ± 0.90^b^	8.93 ± 1.30^b^	9.88 ± 1.42^b^	14.93 ± 1.23^a^
C18 : 1 oleic acid	0.66 ± 0.05^ns^	0.77 ± 0.10^ns^	1.06 ± 0.23^ns^	1.11 ± 0.24^ns^
C18 : 2 *n-6* linoleic acid	24.85 ± 0.95^ns^	23.06 ± 1.45^ns^	24.93 ± 2.48^ns^	24.21 ± 1.02^ns^
C20 : 0 arachidic acid	0.70 ± 0.04^ns^	0.77 ± 0.11^ns^	0.75 ± 0.10^ns^	Nd
C20 : 4 *n-6* arachidonic acid	13.94 ± 1.32^b^	22.62 ± 1.66^a^	23.43 ± 2.14^a^	19.58 ± 2.42^ab^
C20 : 5 *n-3* eicosapentaenoic acid	3.82 ± 0.72^a^	3.05 ± 0.34^ab^	1.73±0.58^b^	Nd
C22 : 5 *n-3* docosapentaenoic acid	0.90 ± 0.26^a^	0.59 ± 0.24^b^	0.32±0.14^b^	Nd
C22 : 6 *n-3* docosahexaenoic acid	6.49 ± 0.47^a^	4.22 ± 0.57^b^	4.33 ± 0.64^b^	2.09 ± 0.15^c^
Total saturated	37.32 ± 1.00^a^	33.16 ± 0.42^b^	33.19±0.77^b^	36.48 ± 1.40^a^
Total unsaturated	62.68 ± 1.00^b^	66.84± 0.42^a^	66.81 ± 0.77^a^	63.52 ± 1.40^b^
Total monoenes	12.69 ± 0.80^b^	12.50 ± 1.51^b^	12.88 ± 1.40^b^	17.65 ± 1.14^a^
Total PUFA *n-3 *	11.21 ± 0.49^a^	7.85 ± 0.54^b^	6.38 ± 1.00^b^	2.09 ± 0.15^c^
Total PUFA *n-6 *	38.79 ± 0.42^bc^	46.49 ± 1.54^a^	47.55 ± 1.17^a^	43.79 ± 1.51^b^
*n-6 : n-3 *ratio	3.49 ± 0.17^c^	6.05 ± 0.54^bc^	8.79 ± 2.16^b^	21.36 ± 1.44^a^
Unsat : Sat	1.69 ± 0.07^ns^	2.02 ± 0.04^ns^	2.02 ± 0.07^ns^	1.76 ± 0.10^ns^
Poly : Sat ratio	1.34 ± 0.05^b^	1.64 ± 0.06^a^	1.63 ± 0.09^a^	1.27 ± 0.08^b^

Values with different superscripts within rows are significantly different at *P* < 0.05; ^ns^no significant difference; ND: not detected.

**Table 3:** Liver tissue fatty acid composition (mg/100 mL; Mean ± SE; *n* = 7) of rats from diffrent treatment groups after 10 weeks of feeding.

Fatty acid composition of sample (mg/100 g)	Group			
	Diet (1 : 1)	Diet (6 : 1)	Diet (30 : 1)	Diet (control)
12 : 0 lauric acid	0.03 ± 0.00^c^	0.22 ± 0.04^a^	0.12 ± 0.04^b^	0.08 ± 0.02^bc^
14 : 0 myristic acid	0.18 ± 0.02^a^	0.18 ± 0.03^a^	0.15 ± 0.02^a^	0.15 ± 0.02^a^
16 : 0 palmitic acid	16.90 ± 0.37^a^	16.61 ± 0.58^a^	16.45 ± 0.56^a^	15.96 ± 0.20^a^
16 : 1 palmitoleic acid	0.35 ± 0.05^a^	0.39 ± 0.09^a^	0.23 ± 0.04^a^	0.21 ± 0.04^a^
18 : 0 stearic acid	21.81 ± 0.87^b^	23.94 ± 0.60^b^	24.22 ± 0.70^a^	24.64 ± 0.38^a^
18 : 1 oleic acid	7.36 ± 0.70^a^	6.20 ± 0.79^b^	6.07 ± 0.32^ab^	5.15 ± 0.68^b^
18 : 2 *n-6* linoleic acid	19.48 ± 1.03^ns^	16.53 ± 0.70^ns^	16.78 ± 1.88^ns^	16.53 ± 0.37^ns^
18 : 3 *n-3* linolenic acid	0.26 ± 0.04^ns^	0.33 ± 0.02^ns^	0.32 ± 0.03^ns^	0.30 ± 0.02^ns^
20 : 0 arachidic acid	0.55 ± 0.04^ns^	0.42 ± 0.06^ns^	0.57 ± 0.07^ns^	0.43 ± 0.05^ns^
20 : 4 *n-6* arachidonic acid	15.58 ± 0.96^b^	20.99 ± 1.09^b^	21.14 ± 0.77^a^	21.74 ± 0.95^a^
22 : 0 behenic acid	0.99 ± 0.08^a^	0.86 ± 0.05^ab^	0.70 ± 0.06^cb^	0.66 ± 0.05^c^
20 : 5 *n-3* ecosapentaenoic	2.73 ± 0.51^a^	0.87 ± 0.17^b^	0.74 ± 0.20^b^	0.73±0.22^b^
22 : 6 *n-3* docosahexaenoic acid	11.98 ± 0.46^ns^	10.26 ± 0.94^ns^	10.51 ± 0.53^ns^	10.72 ± 1.20^ns^
Total saturated	41.44 ± 0.94^ns^	43.29 ± 0.49^ns^	43.21 ± 1.04^ns^	43.56 ± 0.18^ns^
Total unsaturated	58.56 ± 0.94^ns^	56.71 ± 0.49^ns^	56.79 ± 1.04^ns^	56.44 ± 0.18^ns^
Total monoenes	8.53 ± 0.80 ^ns^	7.73 ± 0.84^ns^	7.29 ± 0.48^ns^	6.42 ± 0.62^ns^
Total PUFA *n-3 *	14.97 ± 0.72^a^	11.46 ± 1.08^b^	11.58 ± 0.65^b^	11.75 ± 1.34^b^
Total PUFA *n-6*%	35.06 ± 1.15^ns^	37.52 ± 1.01^ns^	37.92 ± 1.24^ns^	38.27 ± 0.81^ns^
*n-6 : n-3* ratio	2.40 ± 0.20 ^ns^	3.54 ± 0.50^ns^	3.36 ± 0.27^ns^	3.71 ± 0.66^ns^
Unsat : Sat	1.42 ± 0.06^ns^	1.31 ± v0.03^ns^	1.32 ± 0.05^ns^	1.30 ± 0.01^ns^
Poly : Sat ratio	1.21 ± 0.04^ns^	1.13 ± 0.03^ns^	1.15 ± 0.06^ns^	1.15 ± 0.02^ns^

Values with different superscripts within rows are significantly different at *P* < 0.05; ^ns^no significant difference; ND: not detected.

**Table 4:** Adipose tissue fatty acid composition (mg/100 mL; Mean ± SE; *n* = 7) of rats from diffrent treatment groups after 10 weeks of feeding.

Fatty acid composition sample (mg/100 g)	Group			
	Diet (1 : 1)	Diet (6 : 1)	Diet (30 : 1)	Diet (control)
12 : 0 lauric acid	0.06 ± 0.00^b^	0.07 ± 0.00^b^	0.05 ± 0.00^b^	0.08 ± 0.01^a^
14 : 0 myristic acid	1.11 ± 0.02^a^	0.66 ± 0.04^bc^	0.59 ± 0.05^c^	0.77 ± 0.04^b^
16 : 0 palmitic acid	17.67 ± 0.24^bc^	18.69 ± 1.23^ab^	16.05 ± 0.61^c^	20.79 ± 1.01^a^
16 : 1 palmitoleic acid	1.73 ± 0.09^ab^	1.45 ± 0.31^cb^	0.89 ± 0.06^c^	2.23 ± 0.39^a^
18 : 0 stearic acid	4.25 ± 0.08^a^	4.09 ± 0.08^ab^	3.89 ± 0.07^a^	3.95 ± 0.16^ab^
18 : 1 oleic acid	34.61 ± 0.23^ab^	33.52 ± 0.60^cb^	33.04 ± 0.24^c^	35.58 ± 0.73^a^
18 : 2 *n-6* linoleic acid	34.10 ± 0.31^cb^	36.66 ± 1.76^ab^	40.56 ± 0.88^a^	31.94 ± 1.87^c^
18 : 3 *n-3* linolenic acid	0.21 ± 0.01^ns^	0.23 ± 0.01^ns^	0.21 ± 0.02^ns^	0.23 ± 0.01^ns^
20 : 0 arachidic acid	1.95 ± 0.06^ab^	1.89 ± 0.19^b^	2.01 ± 0.03^a^	1.58 ± 0.16^b^
20 : 4 *n-6* arachidonic acid	0.09 ± 0.07^b^	0.67 ± 0.09^a^	0.67 ± 0.03^a^	0.63 ± 0.04^c^
22 : behenic acid	0.19 ± 0.01^b^	0.23 ± 0.02^a^	0.22 ± 0.00^ab^	0.19 ± 0.01^ab^
22 : 1erucic acid	0.03 ± 0.00^a^	0.02 ± 0.01^b^	0.04 ± 0.00^a^	0.02 ± 0.01^b^
20 : 5 *n-3* ecosapentaenoic acid	0.35 ± 0.04^a^	0.09 ± 0.01^b^	0.08 ± 0.02^b^	0.08 ± 0.02^b^
22 : 6 *n-3* docosahexaenoic acid	1.32 ± 0.14^a^	0.41 ± 0.09^b^	0.47 ± 0.08^b^	0.31 ± 0.07^b^
Total saturated	25.98 ± 0.18^a^	26.33 ± 1.14^a^	23.38 ± 0.70^b^	28.23 ± 1.01^a^
Total unsaturated	74.02 ± 0.18^b^	73.67 ± 1.14^b^	76.62 ± 0.70^a^	71.77 ± 1.01^c^
Total monoenes	36.94 ± 0.30^a^	35.61 ± 0.84^cb^	34.63 ± 0.17^ab^	38.57 ± 1.11^a^
Total PUFA *n-3 *	1.88 ± 0.17^a^	0.73 ± 0.11^b^	0.76 ± 0.07^b^	0.62 ± 0.09^b^
Total PUFA *n-6 *	36.19 ± 0.35^b^	37.33 ± 1.78^ab^	41.23 ± 0.87^a^	32.57 ± 1.87^c^
*n-6* : *n-3* ratio	19.50 ± 1.50^b^	59.44 ± 10.49^b^	57.96 ± 6.91^a^	57.33 ± 6.12^a^
Unsat : Sat	2.85 ± 0.03^b^	2.84 ± 0.16^b^	3.30 ± 0.12^a^	2.57 ± 0.13^b^
Poly : Sat ratio	1.43 ± 0.03^b^	1.48 ± 0.12^b^	1.81 ± 0.09^a^	1.20 ± 0.12^b^

Values with different superscripts within rows are significantly different at *P* < 0.05; ^ns^no significant difference; ND: not detected.
